# Time for evidence-based cytology

**DOI:** 10.1186/1742-6413-4-1

**Published:** 2007-01-08

**Authors:** Pranab Dey

**Affiliations:** 1 Department of Cytology, Postgraduate Institute of Medical Education and Research, Chandigarh, India

## Abstract

Evidence-based medicine (EBM) is a fashionable and an extremely hot topic for clinicians, patients and the health service planners. Evidence-based cytology (EBC) is an offshoot of EBM. The EBC is concerned with generating a reproducible, high quality and clinically relevant test result in the field of cytology. This is a rapidly evolving area with high practical importance. EBC is based entirely on research data. The various professional bodies on cytology design and recommend guidelines on the basis of evidences. Once the guideline is implemented and practiced then the experiences of the practicing cytopathologists may be used as a feed back to alter the existing guideline. The various facets of EBC are sampling and specimen adequacy, morphological identification and computer based expert system, integrated reporting, identification of the controversial areas and high quality researches for evidences. It is the duty of the individuals and institutions to practice EBC for better diagnosis and management of the patients. In this present paper, the various aspects of EBC have been discussed.

## Background

Evidence-based medicine (EBM) is a fashionable and an extremely hot topic for clinicians, patients and the health service planners. EBM is defined as "the conscientious, explicit and judicious use of current best evidence in making decision about the care of individual patients [1]. Evidence-based cytology (EBC) is an offshoot of EBM. The EBC is concerned with generating a reproducible, high quality and clinically relevant test result in the field of cytology. This is a rapidly evolving area with high practical importance. To the best of my knowledge, till now there is no article on evidence based cytology in English literature. In this present paper I have discussed the various aspects of EBC.

## Increased demand for EBC

There is increased demand for EBC because of various reasons. There is massive explosion of knowledge in the medical science particularly in basic science. There are also many newer technologies and diagnostic modalities in hand. Therefore there is a need to integrate knowledge. There is also a massive increase of health care costs and overall workload. So it is necessary to make the best use of finite financial resources. The general patients are now more educated and have easier access to electronic media. So, they demand the best quality diagnostic tests in minimal period of time. Lastly there is increasing attention of the medico-legal problems in cytology. Practicing EBC may have some role in these areas.

## EBC practice in clinical cytology laboratory

**Figure 1:**
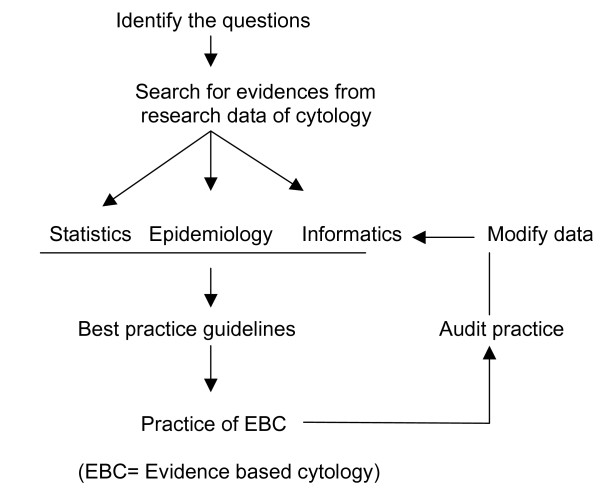
Overview of evidence-based cytology.

Figure 1 highlights the overall view of EBC. There is need of good quality evidences in the field of cytology. These evidences should be derived from high quality research works. The basic data of cytology is handled by three branches of science, 1) statistics 2) epidemiology and 3) informatics. The team of experts should meet together periodically to make guidelines. These guidelines may be considered as the founder pillars of EBC. Based on the guidelines the EBC could be practiced.

## Guidelines in EBC

There are various guidelines developed by different professional bodies for practicing EBC [2-5]. The guidelines may be opinion-based, consensus-based or evidence-based. In opinion based guidelines, one or more experts publish their recommendations which may or may not be opposed by others. In consensus-based guidelines, a group of experts meet and reach to a conclusive guideline. This is relatively rapid, inexpensive method and there may be potential bias in the recommendations [6,7]. Evidence-based guideline is the most desirable one. This is based on systematic identification, critical appraisal, and synthesis of evidences. Ideally, all the recommendations should be supported by enough evidences and the evidences should be analyzed before making guidelines. The most important or controversial questions should be dealt with great attention and systematic review should be done on that aspect. The method section of the guideline should be clearly described by the guideline development group. The member of a guideline development team should be from expert in the field of particular branch of cytology, clinicians, statistician, literature searcher and laboratory manager. The key to successful recommendation of guideline depends on multidisciplinarity of the guideline development team.

A guideline should always be realistic so that its implementation is possible. Evidence based guideline contains graded recommendation which is influenced by the strength of evidence and clinical judgments. If a recommendation is not supported by high grade evidence, then it should be based on consensus of the members [8].

## Seeking evidences for evidence based cytology

Booth et al [9] in a review on evidence based pathology described the methods of evidence seeking techniques in general. However, these could be applied in the field of cytology. The first essential stage in evidence-seeking process is formulating or focusing our question/s. This will help us to use our search strategy on relevant database. Such as the question may be asked as "what will be the best re-screening method in conventional cervical cytology smear?". We can translate this question into a search strategy in the form of key words: "re-screening" AND "method" AND "cervical cytology". If we get systematic review or meta-analysis in the search result, then we can concentrate on these.

We can start with MEDLINE data base as the primary source followed by Cochrane Library and Health Technology Assessment database of the NHS Centre for reviews and dissemination.

## Sampling and specimen adequacy, and EBC

Sampling and specimen adequacy in cytology is an important aspect of EBC because the proper way of sample collection has great impact on the test result. It is also necessary to have adequate cells on the smear. There are various guidelines on the sampling and specimen adequacy by different expert committee [2-5,10,11] on respiratory specimen, urine, thyroid, breast and cervical smears. Table 1 highlights the necessary instructions about the specimen collections in exfoliative cytology. The sensitivity of sputum cytology increases when the samples are obtained over five consecutive days [14,15]. However it has been noted that three consecutive specimens of sputum samples has reasonable sensitivity for detection of malignancies [16]. In microscopy, well preserved, well stained cytologic material with numerous alveolar macrophages are essential for adequate sputum [12]. Greenberg et al noted that adequacy of a sputum sample is directly proportional to the number of alveolar macrophages it contains [17]. Bronchial washings and brushings are complementary to sputum cytology in the diagnosis of respiratory tract lesions. The sample should be obtained from clinically suspicious area by repetitive installation of 3–5 ml sterile balanced salt solution through the bronchoscope. In general a large number of well preserved, optimally stained ciliated bronchial epithelial cells and macrophages are necessary to have a satisfactory specimen. However any specimen that contains cells or agents diagnostic of a pathologic agent should be considered as adequate [3]. The specimens which are heavily contaminated and obscured by oral squamous cells, blood, inflammatory cells or air drying artefacts should be labeled as unsatisfactory [3].

**Table 1:** Sampling and specimen adequacy of exfoliative and gynaecologic sample

Sample	Specimen collection	Adequacy	Diagnostic categorization	Guideline society
Sputum	Early morning spontaneously produced sputum. Three adequate single specimens. [12]	Well preserved, well stained cytologic material with numerous alveolar macrophages. [12]	1. Non-diagnostic 2. Specific benign lesion 3. Atypical cell present, probably benign 4. Atypical suspicious for malignancy 5. Malignancy	Papanicolaou's society guidelines, 1999 [3], [12]
Bronchial wash and brush	Specimen from clinically suspicious area by repetitive installation of 3–5 ml sterile balanced salt solution	A large number of well preserved, optimally stained ciliated bronchial epithelial cells and macrophages.	As above	Papanicolaou's society guidelines, 1999 [3]
Voided urine sample	Any convenient time, 3–4 hours after the patient last voided. approximate 100–300 ml	No definite number of cells mentioned. Probably a slide should contains at least 15 well visualized basal and intermediate cells [13]	1. Negative 2. Infectious agents: bacterial, fungal, viral 3. Nonspecific inflammatory changes: acute inflammation, chronic inflammation, consistent with xanthogranulomatous pyelonephritis 4. Cellular changes associated with chemotherapeutic agents or radiation 5. Epithelial cell abnormalities: a) atypical urothelial cells, b) low grade urothelial carcinoma c) High grade urothelial carcinoma d) squamous cell carcinoma e) adenocarcinoma f) other malignancy 6. Other	Papanicolaou's society guidelines, 2003 [2]

A voided urine specimen can be collected in any convenient time of the day, 3–4 hours after the patient last voided and approximately 100–300 ml of urine should be collected [2]. There is still no specific guidelines on the adequacy of urine sample. Papanicolaou's society of cytopathology practice guidelines task force believe that further studies should be done on adequacy of urine sample [2].

Accurate cervical sampling with the help of correct device/s is the most important part of an adequate cervical smear [18,19]. Inadequate sampling and sample transfer to the glass slides by the traditional or conventional method probably the major cause of false negative cytology [20]. The cytologic sampling of the cervix is taken by different people with different background. When sampling is faulty, the other part of the exercise such as examination and reporting on the specimen are in vain. The medical staff must receive appropriate training in taking cervical sampling. A close monitor of sample adequacy rate for each smear taker and regular feed back may improve the quality of the smear. In addition to properly collected sample, a relevant clinical data from history, inspection and palpation are also important.

The collection devices of cervical smear have great impact on adequacy of the materials [18]. A meta-analysis study showed that the widely used Ayre's spatula is the least effective device for cervical sampling and gives a lower yield of abnormal squamous cells [18]. Extended tip spatula along with cytobrush is the best combination of cervical sampling devise for adequate smear [18].

The Bethesda System, 2001 (TBS) tried to develop a standard framework on evaluation of specimen adequacy and reporting format [21]. According to TBS 2001, satisfactory samples should have the presence of endocervical cells/transformation zone (EC/TZ) component. There should be at least 10 well preserved endocervical or metaplastic squamous cells singly or in groups (table 2) [21]. The assessment of the cellularity of the smears was also reconsidered in the meeting. The minimal squamous cellularity was suggested as 8000 to 12000 well-visualized squamous cells in conventional smears and 5000 squamous cells for liquid based preparations [22]. It seems that the counting of individual cells is clearly impractical. What may be the rationality of the presence or absence of endocervical cells related with sample adequacy? There are conflicting reports on the presence of EC/TZ cells on cervical smears and detection of squamous intraepithelial lesion (SIL) [23-26]. However, many retrospective longitudinal studies have demonstrated that lack of EC/TZ cells on smears do not have increase chance to develop SIL [7,25]. There may be variable presence of endocervical cells in woman who are pregnant, post menopausal or in oral contraceptives [28,29]. We should also remember that the identification of EC/TZ cells is also subjected to inter-observer variability [30]. Nevertheless, the regular feedback on EC/TZ cells may have value in improving overall specimen quality, and awareness of development and use of more sensitive collection technologies. It is expected that the changing incidence of cervical adenocarcinoma may alter the implication of EC/TZ sampling [31]. It is also important to note that other adequacy factors such as obscuring blood or inflammation, and excessive cytolysis should be evaluated in terms of their influence on sensitivity. Liquid based preparations may markedly reduce blood, inflammation or air drying effect along with less number of EC/TZ cells [32]. The number of endocervical cells and sqamous cells for adequacy in liquid based cytology are still a questionable issue.

**Table 2:** Sampling and specimen adequacy of gynaecologic sample

Sample	Specimen collection	Adequacy	Diagnostic categorization	Guideline society
Cervical smear	The whole cervix should be visualized and the whole TZ area should be sampled. Cytobrush along with extended tip spatula is the best combination. (Lancet review). Rotate the spatula through more than one complete turn. Immediately transfer the cells to the slides and immediately fix the smear by immersing in alcohol.	Satisfactory samples should have the presence of endocervical cells/transformation zone component. There should be at least 10 well preserved endocervical or metaplastic squamous cells singly or in groups [22].	• Negative for intraepithelial lesion • Epithelial cell abnormalities Squamous cell • Atypical squamous cells of undetermined significance (ASC-US) and can not exclude HSIL • Low grade squamous intra epithelial lesion (LSIL) • High grade squamous intra epithelial lesion (HSIL) • Squamous cell carcinoma Glandular cell • Atypical glandular cells • Atypical glandular cells favor neoplastic • Endocervical adenocarcinoma in situ • Adenocarcinoma Other • Endometrial cells in a woman > 40 years of age	The 2001 Bethesda System [22]

Criteria for determining adequacy of FNAC of thyroid lesion is not settled till now and it varies from institution to institution (table 3). Papanicolaou Society of Cytopathology Task Forces on Standard of Practice has tried to solve this issue [4] but ultimately did not specify any numbers and groups of thyroid follicular epithelial cells for specimen adequacy. One group of investigators suggest that an adequate sample should contain five to six groups of well-preserved, well-visualized follicular cells with each group contains 10 or more cells [33]. Another group has opinion that ten clusters of follicular cells with at least 20 cells in each cluster are required to have adequate sample [34]. Whereas other suggest that at least six groups of follicular cells should be present on at least two of six aspirates of Fine needle aspiration cytology (FNAC) thyroid for considering adequate specimen [35]. The Papanicolaou Society of Cytopathology Task Forces on Standard of Practice admitted that the cellularity of a specimen is greatly influenced by nature of the lesion. Many cases of benign colloid goiter yield abundant colloid but few follicular cells and should not be considered as inadequate.

**Table 3:** Sampling and specimen adequacy of fine needle aspiration cytology sample

Sample	Specimen collection	Adequacy	Diagnostic categorization	Guideline society
Thyroid	Multiple fine needle aspiration from different sites or fine needle sampling.	Different opinions • Five to six groups of well-preserved, well-visualized follicular cells with each group contains 10 or more cells. [33] • Ten clusters of follicular cells with at least 20 cells in each cluster. [34] • At least six groups of follicular cells should be present on at least two of six aspirates.[35]	• Unsatisfactory for interpretation, specific reason(s) • Benign thyroid nodule/colloid nodule/nodular goiter • Thyroiditis • Cellular follicular lesion, favor non-neoplastic process • Follicular neoplasm, favor benign • Follicular neoplasm, favor malignant • Hurthle cell neoplasm • Malignant specific type if possible • Other	Papanicolaou's society guidelines, 1996 [4]
Breast	Average 2–4 pass in palpable mass More than 2 passes in lesion difficult to stabilize or penetrate, dry tap or in suspected carcinoma.	No consensus on number of cells in a solid breast lesion. Adequacy determined by 1) opinion of the aspirator 2) opinion of the pathologist.	• Benign • Atypical/indeterminate • Suspicious/probably malignant • Malignant: specific types • Unsatisfactory Tumor/nuclear grading should be incorporated in all breast carcinomas whenever possible.	NCI sponsored conference in Bethesda, Maryland 1996 [5]

In September, 1996, the National Cancer Institute sponsored a conference to provide a uniform guideline to FNAC of breast [5]. The controversial topic of adequacy of breast FNA was discussed, but no specific comment was made on requirement for a minimum number of ductal cells for an adequate specimen. (Table 3). The task has been transferred to the aspirator and reporting pathologist to judge the adequacy of the specimen. This particular issue has evoked much controversy in past [36,37]. Many studies have highlighted that the false negative FNAC of breast lesions have a low cellularity [36,38]. Therefore, many authors feel that the sample having minimum number of cells should be reported as non-diagnostic [39,40].

Till now to best of my knowledge, no such guideline has been set for FNAC of lymph node and other organs.

## Identify and interpret: What is the role of expert system?

The tasks of cytopathologist are two folds: at first he should sort out the representative cells or cells of interest and second, he should objectively assess the morphologic alteration of the cells. When the cytopathologist is challenged to detect occasional abnormal cells in the midst of large number of normal cells, computer assisted screening may be helpful. The three different automated screening techniques were developed in the field of cervical cytology. These are PAPNET, AutoPap and AutoCyte Screen [41-46]. However, no such system is available commercially for exfoliative cytology or FNAC smears.

Human perception and interpretation are considered highly valuable in diagnostic interpretation. However it is of no doubt that human assessment of cytology smears and even histopathology sections are too some extent subjective and have very low inter-observer's agreement and low K value in certain situations [47-50]. Various computer based expert systems have been used for an objective approach in diagnosis [51-56]. Expert systems are computer programs that can perform reasoning tasks and can be used for diagnostic difficulties. These systems are based on Bayesian belief network (BBN), artificial neural net (ANN) works, and logistic regression analysis [51-56].

Bayesian belief networks (BBN) have been used by various authors [51,52] to diagnose the cases of benign and malignant lesions in FNAC of breast lesions. The ability of the BBN was satisfactory in diagnosis decision making. It was concluded that this expert system can help in consistent and objective diagnosis of breast lesions.

Another group of researchers [53] investigated the potential of the ANN for the discrimination of benign from malignant breast lesions. This neural network showed excellent performance for correct classification of cells.

The potential of back propagation neural networks in the discrimination of benign from malignant thyroid lesions were examined by Karakitsos P, et al [54]. It was concluded that use of ANN may improve the diagnostic accuracy of FNA of the thyroid gland, especially in cases classified as suspicious for malignancy.

The potential value of morphometry and ANN for discriminating benign from malignant nuclei and lesions of the lower urinary tract on images of routinely processed voided urine smears were investigated by Karakitsos P et al [55]. Application of ANN offered good classification at the nuclear and patient level and promised to become a powerful tool for everyday practice in the cytologic laboratory.

Another group of researchers investigated the potential value of morphometry and the back propagation neural network for the discrimination of benign and malignant lesions in images of routinely processed gastric smears stained by the Papanicolaou technique [56]. Their results indicate that ANN and image morphometry may offer useful information about the potential for malignancy in gastric cells.

This type of expert based system is valuable however the professional societies of the pathologists and cytopathologists should take active participation in designing, evaluating and modifying such systems.

## Reporting

Reporting is one of the important components of generation of a test result. Optimum content and format of the report are necessary for appropriate communication with the clinicians and the patient. There are various guidelines on the diagnostic entities in exfoliative and fine needle aspiration cytology [2-5]. (table 1, 2, 3). Reporting formats can be in the form of check list type i,e proforma report or descriptive report or computer generated. Pathologist should be extremely careful about the terminology or phases to express the certainty. Certain phrases such as "suggestive of", "consistent with", "possibly" etc. should be carefully used. Indiscriminate use of the phrase may generate confusion to the clinician [57].

## Integrated approach of reporting

In recent days, there is massive advancement in the field of molecular biology and computer technology. The massive explosion of knowledge has also significant impact on cytologists. Along with cytomorphology of a lesion, the cytologists now have the information of immunocytochemistry, biochemical profiles and molecular details. So a pan-diagnostic approach is needed [58], which will be in other word a morpho-molecular approach to tissue diagnosis. The cytopathologist will have to combine the clinical history of the disease with the cytomorphology, immunocytochemical findings and molecular details for precise diagnosis. In future, such "molecular cytopathologist" will play a central role in clinical decision making.

## Role of open access article

Open access extends benefits of easy access for the readers and wider real time dissemination of research areas in cytology for the authors-researchers. Some of the benefits of open access and comparison with conventional method of publishing scientific knowledge are depicted in a comic strip published previously [59]. The great benefits of online open access journal are rapid turn around time, real time publications and significant cost savings. The open access journal helps in free flow of scientific information which is immensely important for diagnosis and management of diseases [59]. To avoid publication of misguided substandard or faulty research, peer review of every article is strongly favored [60]. Cytojournal is a peer reviewed cytopathology journal which plays an important role in publishing good quality research data in cytopathology in open access [61]. The research articles are accessible to any one from any part of the world independent of the financial budget of the readers [62]. The online nature allows interactive real time participation. The reader can express their opinion immediately on any article and this has definite positive impact on future of evidence based cytology with wider participation.

## Discussion and conclusion

EBC is based entirely on evidences which should be of good quality. There are many publications which have described how to generate bias free good quality evidences from a research work [63-65]. The good quality research studies can be produced by appropriate study design of the work, explicit identification of the question, appropriate choice of sample population, application of ideal reference standard both to the test and control population, blind comparison of method against reference and outcome, exclusion of confounding variables and introducing reproducible methodology. The ultimate fate of the research work is publication and there may be potential bias in the publication of result because there is greater chance of publication of positive findings [66]. This also should be avoided as far as possible.

The various professional bodies on cytology design and recommend guidelines on the basis of evidences. We also should keep in mind that there is no guarantee that these guidelines will be followed by the practicing cytologists [67]. It is the responsibilities of the individuals or institutions to follow the most accepted guidelines. Once the guideline is implemented and practiced then the experiences of the practicing cytopathologists may be used as a feed back to alter the existing guideline.

Systematic review is also very important in EBC. It is usually moderately comprehensive and summarizes all relevant research information. It identifies various limitations in methodologies of different studies and also helps to limit bias. Systematic review helps to enhance confidence in overall result and also reduces delay between discovery and implementation of the knowledge. At the end it identifies new research questions [68]. However systematic review may provide insufficient data. It is important to find out that whether a comprehensive and explicit search strategy has been used in the review or not? The quality of the included studies in the review is also important.

The other aspect of the EBC is to employ computer based expert system. This is in early stage of development. The professional body of cytology can take part active role for proper designing and application of these systems. Standard algorithm of reporting format may also help in diagnosis [69]. There should be large number of digital pictures available in internet web pages for guidance of diagnosis. Telepathology may also be helpful in diagnosis of difficult cases.

The controversial areas of cytology should be identified and multi-institutional, multinational high quality research works should be done. The result of these researches should be available to all. Open access journals can play an important role for dissemination of knowledge. In fact, there are many articles in Cytojournal on thyroid FNAC, Anal pap, lymph node FNAC, urine cytology, liver etc which probably bears good impact to the cytopathology society [70-74].

It is of no doubt that the use of a diagnostic test is an intervention [75] and the outcome of the cytologic test is part of the decision making process that lead to an improved outcome in clinical context. So we can say that a cytologic test may improve the overall diagnostic process, therapeutic strategy, and economic benefit. In fact it is an integral part of evidence-based medicine. However, it is interesting to note that evidence-based medicine appears to have very little impact in the field of cytology. Probably it is now the ideal time to integrate our information in more productive way for better diagnosis and management of the patients. This is the right time to practice evidence based cytology.

## List of abbreviations used

EBM = Evidence-based medicine

EBC = Evidence-based cytology

TBS = The Bethesda System

EC/TZ = Endocervical cells/transformation zone

FNAC = Fine needle aspiration cytology

BBN = Bayesian belief network

ANN = Artificial neural net

## Competing interests

The author(s) declare that they have no competing interests.
